# Supplementary material for: Mobile Vaccination Teams for Improving Vaccination Coverage in the Kyrgyz Republic: Results of a National Health System-Strengthening Project during the First Two Years of the COVID-19 Pandemic
Source: Children (Basel). 2023 Oct 12;10(10):1681. doi: 10.3390/children10101681 (PMC10605049; doi:10.3390/children10101681)
Supplement: Supplementary file 1 [file children-10-01681-s001.zip › children-2620206-supplementary.pdf]

Supplementary Materials

Mobile Vaccination Teams for Improving Vaccination Coverage in the Kyrgyz Republic: Results of a National Health System-Strengthening Project during the First Two Years of the COVID-19 Pandemic

Table S1. Standards for Reporting Implementation Studies: the StaRI checklist

The primary focus of implementation science is the implementation strategy (column 1) and the expectation is that this will always be completed.	The evidence about the impact of the intervention on the targeted population should always be considered (column 2) and either health outcomes reported or robust evidence cited to support a known beneficial effect of the intervention on the health of individuals or populations.

The StaRI standardsrefers to the broad range of study designs employed in implementation science. Authors should refer to other reporting standards for advice on reporting specific methodological features. Conversely, whilst all items are worthy of consideration, not all items will be applicable to, or feasible within every study.

Checklist item		Reported on page #	Implementation Strategy	Reported on page #	Intervention
			"Implementation strategy" refers to how the intervention was implemented		"Intervention" refers to the healthcare or public health intervention that is being implemented.
**Title and abstract**					
Title	**1**	1	Identification as an implementation study, and description of the methodology in the title and/or keywords		
Abstract	**2**	1	Identification as an implementation study, including a description of the implementation strategy to be tested, the evidence-based intervention being implemented, and defining the key implementation and health outcomes.		
**Introduction**					
Introduction	**3**	1-2	Description of the problem, challenge or deficiency in healthcare or public health that the intervention being implemented aims to address.		
Rationale	**4**	1-2	The scientific background and rationale for the implementation strategy (including any underpinning theory/framework/model, how it is expected to achieve its effects and any pilot work).	6	The scientific background and rationale for the intervention being implemented (including evidence about its effectiveness and how it is expected to achieve its effects).
Aims and objectives	**5**	2	The aims of the study, differentiating between implementation objectives and any intervention objectives.		
**Methods: description**					
Design	**6**	2	The design and key features of the evaluation, (cross referencing to any appropriate methodology reporting standards) and any		

			changes to study protocol, with reasons		
Context	**7**	3	The context in which the intervention was implemented. (Consider social, economic, policy, healthcare, organisational barriers and facilitators that might influence implementation elsewhere).		
Targeted ‘sites’	**8**	3	The characteristics of the targeted ‘site(s)’ (e.g locations/personnel/resources etc.) for implementation and any eligibility criteria.	8, **Supp table S2**	The population targeted by the intervention and any eligibility criteria.
Description	**9**	3	A description of the implementation strategy	6	A description of the intervention
Sub-groups	**10**	NA	Any sub-groups recruited for additional research tasks, and/or nested studies are described		
**Methods: evaluation**					
Outcomes	**11**	**4**	Defined pre-specified primary and other outcome(s) of the implementation strategy, and how they were assessed. Document any pre-determined targets	**Supp table S7**	Defined pre-specified primary and other outcome(s) of the intervention (if assessed), and how they were assessed. Document any pre-determined targets
Process evaluation	**12**	**Table S4**	Process evaluation objectives and outcomes related to the mechanism by which the strategy is expected to work		
Economic evaluation	**13**	NA	Methods for resource use, costs, economic outcomes and analysis for the implementation strategy	NA	Methods for resource use, costs, economic outcomes and analysis for the intervention
Sample size	**14**	NA	Rationale for sample sizes (including sample size calculations, budgetary constraints, practical considerations, data saturation, as appropriate)		
Analysis	**15**	4	Methods of analysis (with reasons for that choice)		
Sub-group analyses	**16**	NA	Any a priori sub-group analyses (e.g., between different sites in a multicentre study, different clinical or demographic populations), and sub-groups recruited to specific nested research tasks		

Table S2. Vaccination schedule in Kyrgyzstan

Birth	2m	3m	5m	9m	12m	24m	6 y	11 y	16 y	26y	36 y	46 y	56 y
HepB	DTP-Hib-HepB	3,5 m - DTP-Hib-HepB	DTP-Hib-HepB	IPV	MMR	DPT	DT	Td	Td	Td	Td	Td	Td
BCG	PCV13	IPV	PCV13		PCV13		MMR	HPV (girls, 2 times)					
	OPV	OPV	OPV										
	RV-5	RV-5	RV-5										

Abbreviations: BCG= antituberculois vaccine; DTP = difteria, tetanus, pertussis vaccine; IPV = inactivated polio vaccine; HepB= hepatitis B vaccine; Hib= Heamophilus Influentiae Type B; HPV= Human papilloma Virus; MMR = measles, mups, rubella vaccine; OPV = oral Polio vaccine; Penta = pentavalent vaccine; PCV13 = Pneumococcal 13 valent vaccine; RV-5 = rotavirus 5-valent vaccine; Td = tetanus difteria vaccine

Table S3. List of settlements covered by MVTs

District	Name of settlements
**Bishkek City**	
Leniniskii	Tynchtyk
	Zher-Yntymagy
Octyabrskii	Altyn- Ordo
Sverdlovskii	Dordoi
	Ak-Jar
Pervomayskii	Kalys-Ordo
**Osh City**	
	Turan
	Kulatova
	Manas- Ata
	Alymbek Datka, Territorial Council №7
	Amur-Timur
**Chui region**	
Moskovskii	Predtechenko
	Besh -Terek
	Malovodnoe
	Tolok
	Kyz-Molo
Issyk-Atinskii	Almaluu
	Yuch-Emchek
	Norus
	Toguz-Bulak
	Karagai- Bulak
	Ichke-Suu
	RotFront
	Ak- Sai
	Telman
	Otogon
Jailskii	Suusamyr
	Transparent
	Birinchi (1) May
	Kojomkul
	Kyzyloi
	Stepnoye
	Kaldyk
	Moltovar
Sokulukskii	Ak- Ordo
	Ata-Jurt
	Tynchtyk
	Erkin-Too , Pole-Chudes
	Kudaibergen
	Akyl-Ordo contour-498, 509, 511
	Altyn- Ordo
	Ak- Ordo 3 ThES
	Mirnoye

	Jany Turmush
	Dacha- Suu
Alamudunskii	Octyabrskoye
	Prigorodnoye
Keminskii	Shabdan
	Orlovka
	Kemin
Panfilovskii	Pervomaiskoye
	Octobrskoye
	Rovnoye
	Chorgolo
Chuiskii	Arpatector
	Sovietskoye
	Kegeti
	Shamsi
	Koshoi
	On-Bir-Jylga
	Madaniyat
	Kosh- Korgon
**Issyk-Kul region**	
Ak-Suiskii	Ak- Bulak
	Ak- Bulun
	Zyndan (Toktogul)
	Tyurgen
	Ak-Chii
	Kyzyl - Kiya (Sovietskoye)
	Kachybek
	Boz-Uchuk (Novovoznesenovka)
	Enchilesh (Boz-Uchuk)
	Ichke-Jergez
	Otradnoye
	Shapak
	Jyrgalan
	Altyn- Arashan
	Enilchek
	Kok- Zhaiyk
	Kok- Kiya
	Ken-Suu
	Koiluu
	Kurgak
Jeti-Oguzskii	Ak-Shyirak
	Uch-Koshkon
	Pogran Zastava
	Pickertick, Yshtyk
	Karakol
	Kara- Sai
	Jeti –Oguz Resort
	Taldy-Bulak
	Podgornoye
	Jyluu- Chuu, Juuk
	Koi-Sary

	Zelenyi Gai
	Dachi
	Pristan, Kainar, Sanatorium
Tonskii	Ala-Bash
	Chetindi
	Kalgar
	Archaly
	Korgonyu Bulak
	Tuura-Suu 2
Tyupskii	Farm №4 - Karkyra
	Farm №3 - Santash
	Chaar-Kuduk
	Chon –Bet
	Sary- Tologoy
	Ken-Suu
	Tabylgyty
	Tasma
	Chon-Toguzbay
	Toktoyan
	Mayak
	Yntymak
Issyk-Kulskii	Chet- Baisoorun
	Kok- Dobo
	Chon-Oruktu
**Naryn region**	
At-Bashinskii	Ak-Muz
	Ak-Moyun
	Ak-Sai Valley
	Kazybek
	Kara-Suu
	Ak-Jar
Ak-Talinskii	Ak- Moynok
	Darbaza
	Arpa
Kochkorskii	Ak-Kaptal
	Iti-Suu
	Karakol
	Sook
	Kalmak -Ashu
	Tyndyuk
Jumgalskii	Sary-Kamysh
Narynskii	Karatal- Zhanyryk
	Jer-Kochku
	Kara-Jylga
	Ak-Kuduk
	Kok- Kiya
	Eki -Naryn
	Jylan-Aryk
**Jalal-Abad region**	
Aksyiskii	Jolbostu
	Kechuu

	Ak- Sai
	Tash-Kuduk
Ala- Bukinskii	Chon-Sai
	Ak- Bashat
	Jetitam
Bazar- Korgonskii	Kyzyl -Unkur
	Kok- Tondu
Nookenskii	Toktonbai
	Shyngyma
	ApyrtaAdyrn
Suzakskii	Jashasyn
	Aral
	Kamysh-Bashy
	Askar- Ata
	Ortok
Toktogulskii	Buurkan
	Kushchu- Suu
	Kotormo
Toguz-Toruskii	Makmal
	Ornok
	Kosh-Bulak
	Karl Marx
	Dodomol
Chatkalskii	Ayakky- Terek
	Myrza-Bulak
	Shamaldy-Sai
Jalal-Abadskii	Ak-Suu
**Osh region**	
Kara –Suuyskii	Chaichi
	Alp-Ordo
	Ak-Terek
	Dostuk
	Langar
	Taldyk
	Teeke
Kara -Kulzhinskii	Kyzyl-Zhar
	Kara -Guz
	Ylai – Talaa
Nookatskii	Kashka- Suu
	Kayindy
	Kara- Bulak
	Kotur-Bulak
	Dary – Suu
	Ak-Terek
	Kapka
	Kara-Koi
	Budaylyk-Chon-Kyshtoo
	Kapchygai
	Syrt- Kyrkol
Aravanskii	Chogom
	Kichik-Alai

	Kara- Bulak
	Too-Moyun
**Talas region**	
Talas	Jegre - Tal
	Ken-Kol
	Besh-Tash
	Kopuro -Bazar
Bakai-Atinskii	Kara-Koyun
Manasskii	Echkilik
**Batken region**	
Kadamjay	
Leylek	
Batken	

Table S4. Rounds of vaccinations performed by MVTs

	Rounds	Dates
**2020**		
1	**First round**	April and May 2020
2	**Second round**	July 2020
3	**Third round**	August 2020
4	**Fourth round**	September 2020
5	**Fifth round**	Novembre 2020
6	**Sixth round**	Decembre 2020
**2021**		
1	**First round**	March 2021
2	**Second round**	June 2021
3	**Third round**	June 2021
4	**Fourth round**	August 2021
5	**Fifth round**	October 2021
6	**Sixth round**	November 2021

Table S5. Coverage of hard-to-reach settlements by regions and MVTs rounds (2021)

No.	Region/City	Round 1	Round 2	Round 3	Round 4	Round 5	Round 6
1	Bishkek	68.8%	70%	85%	94%	99%	99%
2	Osh	80%	80%	78.9%	85.8%	95.7%	86%
3	Chui region	100%	100%	100%	100%	100%	100%
4	Issyk-Kul region	73%	73%	73%	73%	73%	78%
5	Naryn region	60%	38.2%	38.2%	40.2%	47.3%	65.2%
6	Jalal-Abad region	58%	78%	79%	89%	55%	30%
7	Osh region	56%	47%	54%	50.1%	52.8%	88%
8	Talas region	100%	100%	100%	100%	100%	100%
9	Batken region	80%	70%	70%	90%	100%	59%

Table S6. Number of people vaccinated, by vaccine, year 2020

Vaccinations	Batken region	Naryn region	Jalal-Abad	Chui region	Issyk-Kul	Talas region	Bishkek city	Osh city	Osh region	Total
bOPV - 1 (oral polio)	432	425	1529	1445	560	451	580	402	1111	6935
bOPV - 2 (oral polio)	363	375	1298	900	573	492	638	346	907	5892
bOPV - 3 (oral polio)	312	357	1509	1137	573	493	582	328	969	6260
**Total**	1107	1157	4336	3482	1706	1436	1800	1076	2987	19087
IPV (inactivated polio)	447	288	1296	1132	541	487	577	326	888	5982
Penta - 1 (DTP-HBV-Hib)	538	417	1539	1331	573	443	760	400	1111	7112
Penta - 2 (DTP-HBV-Hib)	472	385	1309	861	581	483	586	346	896	5919
Penta - 3 (DTP-HBV-Hib)	422	401	1477	1040	551	475	566	328	975	6235
**Total**	1432	1203	4325	3232	1705	1401	1912	1074	2982	19266
RV-1	515	344	1462	1185	553	418	510	382	1099	6468
RV-2	448	333	1248	815	539	464	669	388	875	5779
RV-3	364	282	1289	1072	583	456	498	256	993	5793
**Total**	1327	959	3999	3072	1675	1338	1677	1026	2967	18040
MMR at 1 year	483	639	1310	1590	1000	555	875	388	925	7765
MMR at 6 years	351	685	892	1261	842	808	580	302	1134	6855
**Total**	834	1324	2202	2851	1842	1363	1455	690	2059	14620
PCV - 1 (Pneumococcal)	537	420	1490	1235	573	441	559	392	1093	6740
PCV - 2 (Pneumococcal)	431	391	1460	985	570	473	532	322	893	6057
PCV - 3 (Pneumococcal)	491	608	1379	1578	742	560	832	396	960	7546
**Total**	1459	1419	4329	3798	1885	1474	1923	1110	2946	20343
DPT-2 years	477	780	952	1303	786	431	861	560	992	7142
Dt- 6 years	302	730	1117	1355	812	810	551	302	1134	7113
**Total**	687	1331	1915	2219	1503	1241	1375	862	2126	13259

Td - 11 years	624	703	748	2609	846	863	83	778	1020	8274
Td- 16 years	504	553	733	1758	507	475	125	472	1291	6418
Td -26 years	269	364	548	1005	529	369	480	550	1327	5441
Td- 36 years	249	345	373	973	371	321	566	530	1141	4869
Td- 46 years	185	269	408	757	377	324	356	408	909	3993
Td- 56 years	279	576	578	969	522	230	232	368	892	4646
**Total**	13476	3337	4827	9370	4223	3823	3151	3968	8706	54881
Number of mobile teams involved	5	6	ten	ten	5	5	7	5	eleven	64
Total vaccinations given in the Republic										**165478**
**Children under 1**										**90483**
**Children under 16**										**125289**
Adults										**18949**

Abbreviations: DTP= difteria, tetanus, pertussis vaccine; IPV= inactivated polio vaccin; MMR=measles, mups, rubella vaccine; OPV= oral Polio vaccine; Penta= pentavalent vaccine; PCV= Pneumococcal vaccine; RV=rotavirus vaccine ;Td= tetanus difteria vaccine

Table S7. Number of people vaccinated, by vaccine, year 2021

Vaccinations	Batken region	Naryn region	Jalal-Abad	Chui region	Issyk-Kul	Talas region	Bishkek city	Osh city	Osh region	Total
**bOPV - 1 (oral polio)**	494	555	1139	1664	627	340	854	656	1966	8295
**bOPV - 2 (oral polio)**	436	473	939	1478	509	439	928	619	1780	7601
**bOPV - 3 (oral polio)**	356	494	1044	1900	483	384	1031	556	1749	7997
**Total**	1286	1522	3122	5042	1619	1163	2813	1831	5495	23893
**IPV (inactivated polio)**	1171	454	1078	4945	531	513	1145	1442	1721	13000
**Penta - 1 (DTP-VGV-Hib)**	490	560	1982	1549	608	340	827	660	1971	8987
**Penta - 2 (DTP-VGV-Hib)**	436	467	932	1396	494	428	890	574	1791	7408
**Penta - 3 (DTP-VGV-Hib)**	418	500	1814	1926	464	357	893	550	1770	8692
**Total**	1344	1527	4728	4871	1566	1125	2610	1784	5532	25087
**RV-1**	474	502	1072	1306	611	316	852	647	1927	7707
**RV-2**	427	465	929	929	507	408	891	590	1746	6892
**RV-3**	409	495	1057	1178	477	324	899	449	1537	6825
**Total**	1310	1462	3058	3413	1595	1048	2642	1686	5210	21424
**MMR at 1 year**	569	632	1896	1857	856	986	927	646	1853	10222
**MMR at 6 years**	519	661	899	1757	690	1177	740	405	1847	8695
**Total**	1088	1293	2795	3614	1546	2163	1667	1051	3700	18917
**PCV - 1 (Pneumococcal)**	502	489	1175	1738	615	337	853	649	1953	8311
**PCV - 2 (Pneumococcal)**	451	481	1149	1828	461	362	881	555	1767	7935
**PCV - 3 (Pneumococcal)**	523	620	1100	2188	724	979	943	789	1751	9617
**Total**	1476	1590	3424	5754	1800	1678	2677	1993	5471	25863
**DPT-2 years**	555	713	912	1421	621	367	1072	611	1882	8154
**Dt- 6 years**	517	638	905	1682	641	1170	919	398	1868	8738
**Total**	1072	1351	1817	3103	1262	1537	1991	1009	3750	16892
**Td- 11 years**	345	517	672	2779	468	484	482	286	1258	7291

**Td- 16 years**	297	496	557	2231	302	365	266	265	901	5680
**Td - 26 years**	163	225	538	928	148	333	662	391	558	3946
**Td- 36 years**	163	216	394	862	151	275	687	343	536	3627
**Td - 46 years**	102	224	361	833	134	292	487	286	399	3118
**Td - 56 years**	96	229	329	607	118	200	280	201	363	2423
**Total**	1166	1907	2851	8240	1321	1949	2864	1772	4015	26085
Number of mobile teams involved	5	7	10	10	6	5	14	9	12	78
Total vaccinations in the Republic										171161
**Children under 1**										**109267**
**Children under 16**										**158047**
Adults										**13,114**

Abbreviations: DTP= difteria, tetanus, pertussis vaccine; IPV= inactivated polio vaccin; MMR=measles, mups, rubella vaccine; OPV= oral Polio vaccine; Penta= pentavalent vaccine; PCV= Pneumococcal vaccine; RV=rotavirus vaccine ;Td= tetanus difteria vaccine

Table S8. Contribute of MVTs to the total immunization coverage in the country (2021)

Vaccination	Batken region	Naryn region	Jalal-Abad	Chui region	Issyk-Kul region	Talas region	Bishkek city	Osh city	Osh region	Total
**bOPV - 3 (oral polio)**	2.7%	9.2%	3.5%	9.4%	5.4%	7.6%	4.5%	6.5%	5.5%	5.5%
**IPV (inactivated polio)**	9.0%	8.5%	3.7%	24.4%	5.9%	10.1%	5.0%	16.9%	5.4%	8.9%
**Penta - 3 (DTP- HBV -Hib)**	6.5%	1.5%	0.0%	4.0%	1.0%	3.2%	0.2%	1.0%	1.1%	1.7%
**RV-3**	3.1%	9.2%	3.6%	5.8%	5.3%	6.4%	3.9%	5.3%	4.8%	4.7%
**MMR at 1 year of age**	4.4%	11.8%	6.4%	9.2%	9.6%	19.5%	4.0%	7.6%	5.8%	7.0%
**MMR at 6 years**	3.7%	11.3%	3.1%	7.7%	6.9%	21.3%	3.8%	4.5%	5.6%	5.8%
**PCV - 3 (Pneumococcal)**	4.0%	11.5%	3.7%	10.8%	8.1%	19.3%	4.1%	9.2%	5.5%	6.6%
**DPT-2 years**	4.0%	12.9%	3.1%	6.4%	6.3%	6.9%	4.6%	6.6%	5.5%	5.3%
**DT- 6 years**	3.6%	10.9%	3.1%	7.4%	6.4%	21.2%	4.7%	4.4%	5.6%	5.8%
**Td- 11 years**	2.9%	9.0%	2.6%	13.4%	4.6%	8.6%	2.4%	3.8%	4.5%	5.4%
**Td - 16 years**	3.4%	10.0%	2.8%	14.2%	3.7%	8.3%	1.7%	5.0%	4.3%	5.5%
**Td - 26 years**	1.8%	7.2%	2.8%	6.7%	2.4%	10.3%	6.0%	6.7%	2.7%	4.3%
**Td - 36 years**	2.1%	7.5%	2.5%	6.3%	2.7%	10.0%	6.4%	7.3%	3.2%	4.5%
**Td- 46 years**	2.0%	8.8%	3.2%	7.8%	3.0%	13.1%	5.5%	8.5%	3.1%	5.1%
**Td - 56 years**	2.2%	10.4%	3.4%	5.9%	2.8%	11.1%	4.2%	6.8%	3.1%	4.5%

Abbreviations: DTP= difteria, tetanus, pertussis vaccine; IPV= inactivated polio vaccin; MMR=measles, mups, rubella vaccine; OPV= oral Polio vaccine; Penta= pentavalent vaccine; PCV= Pneumococcal vaccine; RV=rotavirus vaccine ;Td= tetanus difteria vaccine
